# A Comparison of Machine Learning Approaches to Activity Classification Using IMU Data Collected in a Community-Based Setting

**DOI:** 10.3390/s26175357

**Published:** 2026-08-25

**Authors:** Hans E. Anderson, Robert A. Scheidt, Kimberly D. Bassindale

**Affiliations:** 1 Department of Orthopaedics, Division of Physical Medicine and Rehabilitation, Stanford Medicine, Stanford, CA 94305, USA; 2 Joint Department of Biomedical Engineering, Marquette University and Medical College of Wisconsin, Milwaukee, WI 53233, USA; 3 Department of Physical Therapy and Human Movement Sciences, Northwestern University, Chicago, IL 60611, USA; kimberly.bassindale@northwestern.edu

**Keywords:** machine learning, activity classification, inertial measurement units, wearable technology, mobile health

## Abstract

**Highlights:**

**What are the main findings?**

- Several classical machine learning models outperformed two deep learning models (as well as a chance-level “dummy” classifier) in classifying functional activities using both a small and an expanded set of features derived from an IMU dataset from four wearable sensors.

**What are the implications of the main findings?**

- Functional activity classification using IMU data from bilateral wrist and ankle sensors is feasible in a less constrained, community-based setting.
- Multiple ML models performed with balanced accuracy >60% (far better than chance performance) despite a small, heterogeneous dataset and short (5 s) input windows, supporting their potential use in small-sample clinical populations.
- For small-sample human activity recognition (HAR), model choice, feature complexity, non-target (“null”) activity handling, and sensor layout should be evaluated comprehensively in heterogeneous datasets for real-world applications.

**Abstract:**

Machine learning (ML) algorithms can be used to extract clinically meaningful information from movement data captured by inertial measurement units (IMUs), but many human activity recognition (HAR) pipelines are developed on large laboratory datasets that may not reflect small, heterogeneous, real-world samples. The purpose of this study is to systematically compare the accuracy of multiple ML models, feature sets (both simple and expanded), class balancing strategies, null and transition period handling techniques, and sensor configurations for recognizing a set of four everyday activities extracted from IMU time series data from an age-diverse population. Six ML classifiers were trained and tested: multilayer perceptron, random forest, k-nearest neighbors, logistic regressor, CatBoost, and gaussian naive bayes. These approaches were used in a pipeline with differing sampling techniques including the synthetic minority oversampling technique or random undersampling, and feature handling steps including principal component analysis or a Select-From-Model metatransformer. Additionally, two deep learning methods, DeepConvLSTM and ResGCNN, were trained and tested. Accuracy, precision, recall, and area under the receiver operating characteristic curve were compared to a dummy classifier as a benchmark approximation to chance performance. All pipelines performed better than the dummy classifier, with model accuracy ranging between 0.427 and 0.644. This study demonstrated the ability of several ML algorithms to properly recognize a set of functional activities using limited IMU data from both children and adults.

## 1. Introduction

Wearable sensors and machine learning (ML) algorithms are finding increasing acceptance in applications related to physical rehabilitation [1]. Inertial measurement units (IMUs) are among the most frequently used sensors for these purposes and have been deployed to address diverse diseases such as stroke, spinal cord injury, and Parkinson’s disease [1]. ML algorithms allow for the transformation of IMU time series data into meaningful information in applications such as activity detection, activity recognition, and clinical assessment [2]. For example, IMU data can be used variously to quantify step count or duration of walking bouts (activity detection), or to assess gait quality in terms of temporo-spatial gait parameters and joint kinematics (activity recognition/clinical assessment) [3].

Many different ML algorithms have been employed to interpret data collected from IMUs in the context of rehabilitation, including tree-based methods, neural networks, linear methods, and ensemble approaches [2]. The approaches can differ in terms of accuracy performance and can require different amounts of computing resources, ranging from requiring few (e.g., only on-device processing) to many (e.g., the use of cloud computing); the tradeoff between performance and computing power may influence algorithm choice in any given application [4]. For example, naïve Bayes methods, support vector machines, and decision trees are particularly useful in low power settings [5], but may underperform advanced techniques when more resources are available. Furthermore, dataset size is another key consideration for model selection, since traditional machine learning methods, like tree-based methods, tend to generalize more effectively from smaller datasets, whereas deep learning architectures such as DeepConvLSTM typically require substantially larger volumes of training data to reach their performance potential [6]. This distinction holds significant implications in rehabilitation research, where data collection from clinical populations is often constrained by small cohort sizes. Furthermore, much of the existing literature has applied human activity recognition (HAR) techniques to larger, well-controlled datasets curated from relatively homogeneous populations in laboratory settings (e.g., OPPORTUNITY [7], PAMAP2 [8], WISDM [9], and UCI-HAR [10]). Consequently, HAR algorithms derived from lab-based datasets may reflect ideal conditions that may not translate well to smaller datasets or real-world settings. For example, Stojchevska et al. (2023) demonstrated that models trained on MHEALTH, a large publicly available IMU dataset, performed poorly when applied to real-world data and required further personalization training to improve accuracy [11]. Developing activity recognition pipelines that perform reliably under less controlled, data-limited conditions is therefore a practical and clinically meaningful priority.

Heterogeneity remains another major challenge for HAR model development because many clinical populations, such as individuals with stroke or cerebral palsy, present with greater movement variability compared to healthy individuals [12,13,14]. Additionally, factors like age [15], body size [16], and functional ability [17] can influence movement patterns and, therefore, the IMU signals used for HAR, resulting in potentially greater intraclass variability. One simple strategy for addressing this variability is to develop separate models for distinct populations. For example, many HAR models have been developed independently for adults [5] or children [18,19], reflecting the limited generalizability of models across populations in the absence of personalization or transfer learning (c.f., [20]). In some cases, the delineation between population subgroups may be well defined, but for known heterogeneous populations, such as in the case of stroke, subgrouping may be less practical and may risk overfitting. Likewise, many HAR studies evaluate activities with stereotyped movement patterns, such as walking, sitting, stair climbing, or lying down, while less attention has been given to distinguishing activities that require similar limb movement patterns or to activities that inherently have greater interpersonal variability in a real-world environment (e.g., dancing).

Another limitation of current HAR research is that methodological decisions are often evaluated independently. Previous studies have investigated individual classifiers [6,21,22], feature extraction methods [23,24], or sensor placement [19,22,25,26], but relatively few have systematically examined how these design choices interact when data are both heterogeneous and limited. As a result, researchers developing HAR systems for smaller datasets have limited evidence to guide collective decisions regarding feature complexity, class balancing, dimensionality reduction, treatment of transition periods and null activity, or the number and placement of wearable sensors.

The present study addresses these gaps through an extensive methodological evaluation of wearable HAR using a small, heterogeneous convenience sample dataset. We aim to systematically compare the accuracy of multiple ML models, feature sets (both simple and expanded), class balancing strategies, transition period handling techniques, and sensor configurations. The approach is motivated by a broader desire to gain insight into best practices for using wearable technology and an edge computing platform to perform real-time activity recognition over a set of everyday activities extracted from IMU time series data collected from an age-diverse population. The selected activities were designed to include activities of daily living that are often the target of therapeutic training, while also reflecting inter-individual variability across a diverse cohort of healthy participants drawn from a community-based setting that may better reflect real-world scenarios. We hypothesize that meaningful activity recognition is feasible despite the substantial constraints imposed by a dataset defined by its small size and heterogeneous participant pool. Moreover, we expect that while many ML approaches will predict activity type with greater accuracy compared to chance probability, one of the approaches will be the unambiguous top performer.

## 2. Materials and Methods

Data were collected from a convenience sample of 23 community members (16 female, age range: 3–68) who participated in this study, which took place during the 2-day 2023 Maker Faire Milwaukee expo. All procedures received institutional review and approval from Medical College of Wisconsin and Marquette University (HR-4500 on 18 September 2023). All participants under the age of 18 (*n* = 14) provided written, informed assent with guardian consent. The remaining participants aged 18 or older (*n* = 9) provided written, informed consent in accordance with the Declaration of Helsinki (2013 revision).

### 2.1. Materials

Four MetaMotion R+ activity monitors (MBIENTLAB; San Francisco, CA, USA) were used for data collection; the devices include a 3-axis accelerometer (range: ±16 g), and 3-axis gyroscope (range: ±2000°/s). Accelerometer and gyroscope data were sampled at 50 Hz; time-stamped data were streamed to an iOS device equipped with the commercially available mobile application MetaBase (MbientLab Inc., San Jose, CA, USA).

### 2.2. Protocol

**Figure 1:**
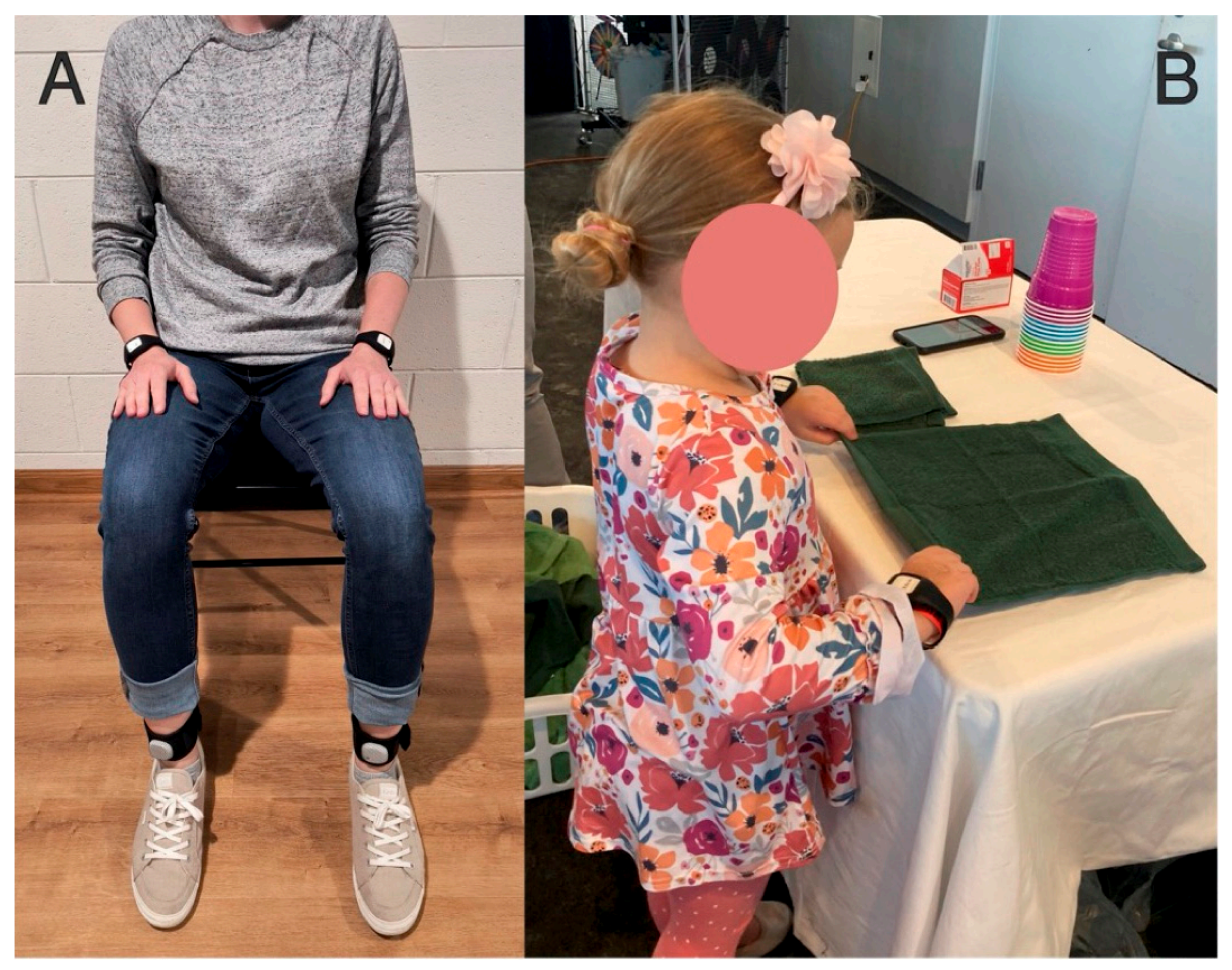
(**A**) MetaMotionR+ devices worn on both wrists (housed in silicone bands) and both ankles (housed in neoprene bands), and (**B**) a young participant completing the towel folding activity with ongoing IMU data collection.

Each of the activity monitors were housed in neoprene or silicone bands and worn on each wrist and ankle while the participant was seated comfortably (Figure 1A). We positioned the four wearable sensors on the bilateral wrists and ankles to capture both upper and lower extremity movement while maintaining a setup that can be practically implemented in community-based populations consistent with the setting of our study. These sensor locations are also well-established in prior work [1,2,3], having demonstrated strong performance for activity recognition using machine learning approaches.

IMU data were gathered while participants completed a series of four functional activities performed in the following order: (1) folding towels; (2) stacking cups into a pyramid; (3) standing up and sitting down from a chair (sit-to-stands); and (4) dancing to a song of choice from the Nintendo Wii videogame “Just Dance” (Ubisoft, Saint-Mandé, France, 2009) (Figure 1B). These activities were chosen for multiple reasons. First, the tasks of folding towels and stacking cups were chosen as representative of bimanual upper-extremity activities with similar structural demands, allowing us to evaluate the algorithms’ abilities to differentiate between tasks that share overlapping movement patterns. The sit-to-stand task was included as a functional, whole-body movement to provide contrast with the upper-extremity tasks. Lastly, the dance activity was chosen to examine whether the algorithms could identify activities characterized by high inter-individual variability, as movement patterns during dance are inherently influenced by personal style and preference. We asked participants to perform each activity for 60 s. Rest intervals were provided between target activities and typically lasted from 10 to 30 s but could be extended (2 to 3 min) if the participant so desired. Activity periods were timed with a stopwatch and transition times between the activities and rest were documented by the experimenter for later use in segmenting the data time series. Instructions for each of the activities were straightforward (e.g., “sit down and stand up from this chair at a comfortable pace”), enabling participants to select a strategy that felt most natural to them.

### 2.3. Data Preprocessing

Each MetaMotionR+ device generated time-stamped accelerometry values for the {X, Y, Z} axes in gravity units (1 g = 9.8 m/s^2^) and gyroscope angular velocity values about the {X, Y, Z} axes in °/s. Data from the four devices were synchronized by resampling at 50 Hz to a common time base.

**Figure 2:**
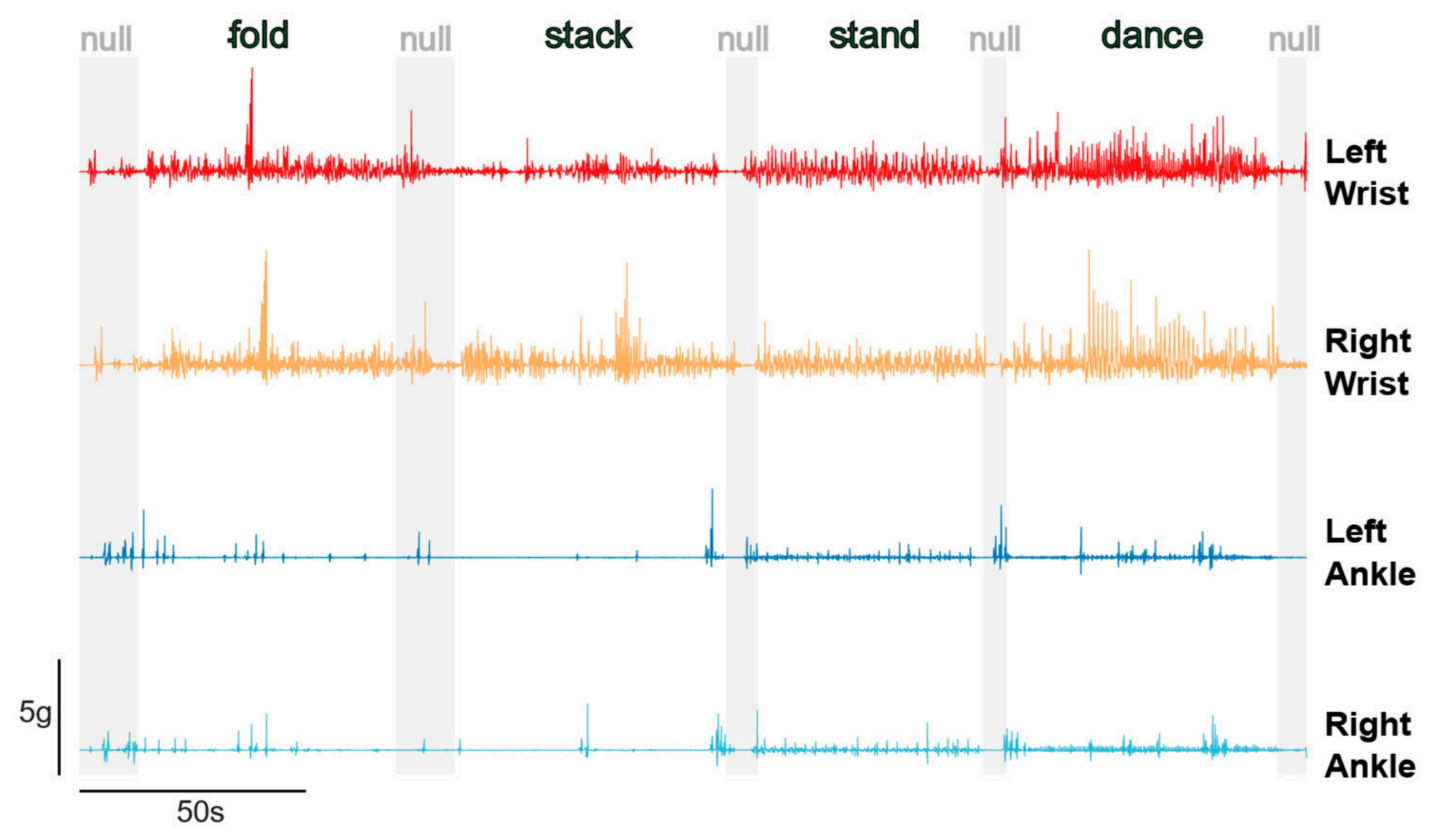
Sample of acceleration magnitude (g) over time between activities: folding towels (fold), stacking cups (stack), sit-to-stand (stand) and dance. “Null activity” periods are highlighted in grey.

Raw time-domain data were bandpass filtered between 0.25 and 2.5 Hz as recommended by Bailey, Klaesner, and Lang (2014) [27] to accentuate IMU signal components related to purposeful limb movements. Acceleration magnitude (Figure 2) and angular velocity magnitude were each calculated using the Euclidean norm (root mean square of the filtered X-, Y-, and Z-axes); the resulting scalar values were then integrated within each 1 s epoch. We extracted several time-domain features for input into ML algorithms using the scalar acceleration and angular velocity magnitude signals from each successive 5-epoch window of synchronized kinematic activity from each device. Time-domain features included: the mean, standard deviation, root mean square (RMS), first quartile, third quartile, median, maximum, and minimum of the acceleration and gyroscope magnitude signals.

We also extracted frequency-domain features from the raw time series data within each 5 s window of activity. Frequency-domain features were derived from the power spectral density applied to unfiltered acceleration and gyroscopic magnitude signals. Frequency-domain features included: the signal power computed within equal-width low- (1–9 Hz), medium- (9–17 Hz) and high-frequency (17–25 Hz) bands. This choice was motivated by observations that the majority of signal power in the kinematics of typical unconstrained human movements of the upper and lower extremities is band-limited to frequencies less than about 9 Hz (see [28,29]), whereas signal energy in the higher-frequency bands captures faster movement dynamics or harmonics, and may be able to distinguish between upper-extremity movements involving fine manipulation of objects (e.g., the Nine Hole Peg Test [29]) vs. those involving whole arm motions (such as a drawing task) [29]. The 5 s window width was chosen to provide sufficient data for both time- and frequency-domain features while maintaining temporal resolution to facilitate responsiveness of real-time activity classification. All data preprocessing was performed in MATLAB R2022B 9.13.

### 2.4. Machine Learning

**Figure 3:**
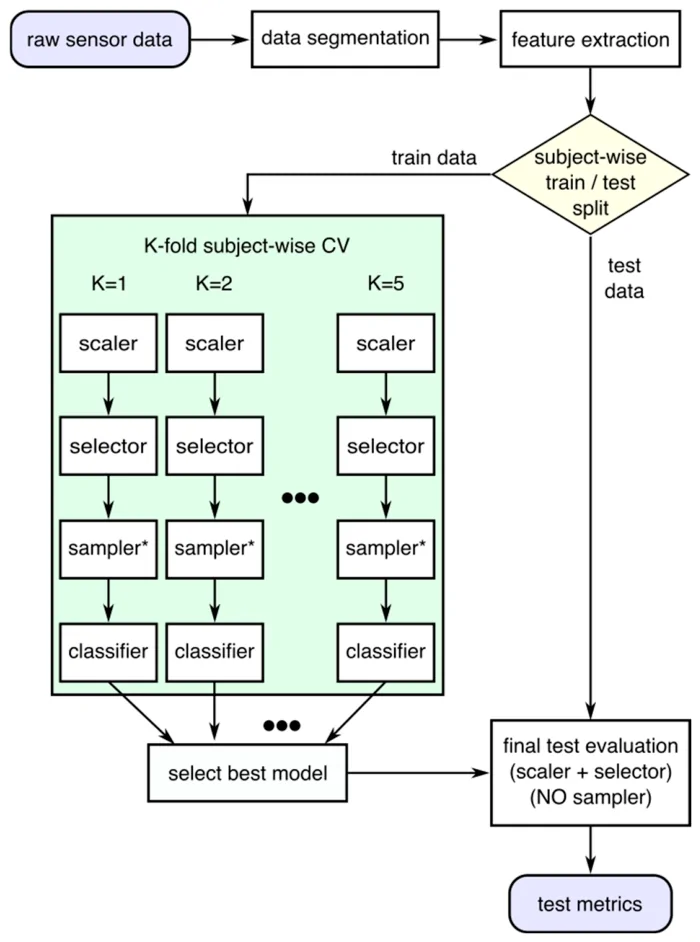
Flowchart of data processing, model training, and evaluation procedures. K-fold subject-wise cross validation (CV; K = 5) was used to identify the best ML model for the therapeutic activity recognition problem described in the manuscript. (* The sampling step was skipped during validation.).

We approached the activity recognition task as an ML classification problem. To address sampling bias in the dataset, which favored younger participants, participants were divided into two age groups: ≤12 years or >12 years. These two groups were split into train and test groups with an approximate 70:30 ratio. Subsequently, the two train groups were combined into a single train group and the two test groups were combined into a single test group. Data from participants were scaled using scikit-learn’s StandardScaler. Six ML classifiers were trained and tested: multilayer perceptron (MLP), random forest (RF), k-nearest neighbors (KNN), logistic regressor (LR), CatBoost, and gaussian naive bayes (GNB). To address imbalances in the number of instances within each activity class, which can cause bias during training, we included the random undersampling (RUS) or the synthetic minority oversampling technique (SMOTE) in the model pipeline. We then either trained the model without prior feature handling or performed feature handling to reduce dimensions or features with principal component analysis (PCA) or a Select-From-Model (SFM) metatransformer. To benchmark the above models against an approximation of chance (i.e., random) classification, we also trained a dummy classifier, which creates class predictions independent of input features, using equal weightings for classes. We also compared the results to two deep learning methods, DeepConvLSTM and Res-GCNN, which used the post-filtering, pre-feature creation raw data stream. We selected DeepConvLSTM based on its prior use in HAR and its demonstrated ability in edge computing scenarios [30]. We selected ResGCNN based on its ability for few shot learning in HAR [31]. To further enhance the performance of the deep learning methods, data augmentation using jitter, time-warp, scale, and rotate techniques was completed. We also used the Python Optuna library to attempt to improve the speed of the grid search for ResGCNN and DeepConvLSTM. We used a grid search of the hyperparameters for each model with subject-wise 5-fold cross-validation to optimize and train the models using balanced accuracy (i.e., macro recall) for scoring (Figure 3; see also Supplementary File S1).

Model performance was subsequently evaluated using the test dataset and quantified using five performance measures:

- *Accuracy*: The number of correct predictions divided by the total number of predictions.

(1) $$Accuracy=\frac{number\;of\;correct\;predictions}{total\;number\;of\;predictions}$$

- *Precision* for a given class (*positive predictive value*): The fraction of correctly predicted members of a class (true positives, *TP*) normalized to the total predicted members of that class (sum of true positives and false positives, *FP*).

(2) $${Precision}_{class}=\frac{{TP}_{class}}{{TP}_{class}+{FP}_{class}}$$

- *Recall* for a given class (aka *sensitivity* or *true positive rate*): The fraction of positives predicted in a class (true positives) normalized to the total number in that class (sum of the true positives and the false negatives, *FN*).

(3) $${Recall}_{class}=\frac{{TP}_{class}}{{TP}_{class}+{FN}_{class}}$$

- The area under the receiver operating characteristic curve (*AUC*): The area underneath the curve obtained by plotting the true-positive rate (same as recall), and the true-negative rate (the fraction of true negatives of the total number of negatives) given different cutoff thresholds for a binary classifier. A one-vs-one method (with each class compared to each of the other class) is used to obtain the *AUC* of each class.

(4) $$Macro\;Average=\frac{sum\;of\;score\;metric\;for\;all\;classes}{total\;number\;of\;classes}$$

- *F1 score*, or the harmonic mean of precision and recall, was also calculated because it is a more robust measure of performance when class imbalance is suspected (Equation (5), where *P* represents precision, and *R* represents recall).

(5) $$F_{1}=\frac{2\cdot P\cdot R}{P\;+\;R}=\frac{2\cdot TP}{2\cdot TP+FN+FP}$$

To assess overall recall, precision, AUC, and F1 score metrics for each model, the class values were each macro-averaged by dividing the sum of the score metric for all classes by the total number of classes.

To evaluate the trade-off between model complexity and computational cost, we compared model performance using (i) a reduced feature set consisting of basic time-domain features (mean, standard deviation, and RMS) for each sensor, and (ii) the “expanded” feature set including all of the time-domain features (i.e., mean, standard deviation, RMS, first quartile, third quartile, median, maximum, and minimum) and frequency-domain power spectral density features (i.e., the signal power computed within low-, medium- and high-frequency bands). This comparison was motivated by the goal of developing an activity recognition algorithm suitable for deployment on devices with limited computational power.

Statistical testing between models to evaluate differences in model performance was undertaken using the bootstrap difference test with significance set to 0.05. 95% confidence intervals (CIs) were calculated by using bootstrapping with *n* = 1000. All ML was undertaken using Python (ver. 3.13), scikit-learn (ver. 1.1.2), CatBoost (ver. 1.2.10), PyTorch (ver. 2.2.2) and imbalanced-learn libraries (ver. 0.10.1). All machine learning was completed on a MacBook Pro with a 2.8 Ghz Quad-core Intel Core i7 with 16 GB 2133 MHz LPDDR3 RAM running macOS 15.6.

## 3. Results

### 3.1. Study Population

This community-based convenience sample of participants was predominantly female and white, with a wide range of ages (median: 12 years; see Table 1). Participant heights were centered about 60 inches, and their weights centered about 103 lbs. See Supplemental Table S3 for individual participant demographics.

Each participant was instructed to perform each of the four target activities for 60 s and then rest quietly for a brief period of time before starting the next activity. As can be seen for a selected participant in Figure 2, participants did not always comply with the request to rest quietly between activities. As such, we refer to the data collected in the periods between instructed activities as “null class” activity data. The duration of rest periods varied both within and across participants, such that the distribution of 5 s data windows (samples) favored the null class (30.4%, Table 2).

### 3.2. Model Performance with Expanded Feature Set

All tested algorithms yielded classification performance well above that of chance, as represented by the dummy classifier, when we used the expanded feature set as inputs to the models (Table 3). A CatBoost classifier was found to perform best, with accuracy, precision, recall, and macro-averaged F1 values all approximately 64% (Table 3), although all but GNB and DC exhibited very similar performance. CatBoost, MLP, RF, KNN, and LR all had AUCs greater than 0.8. As expected, the dummy classifier (feature-independent or random class assignment) performed poorly across all metrics.

**Figure 4:**
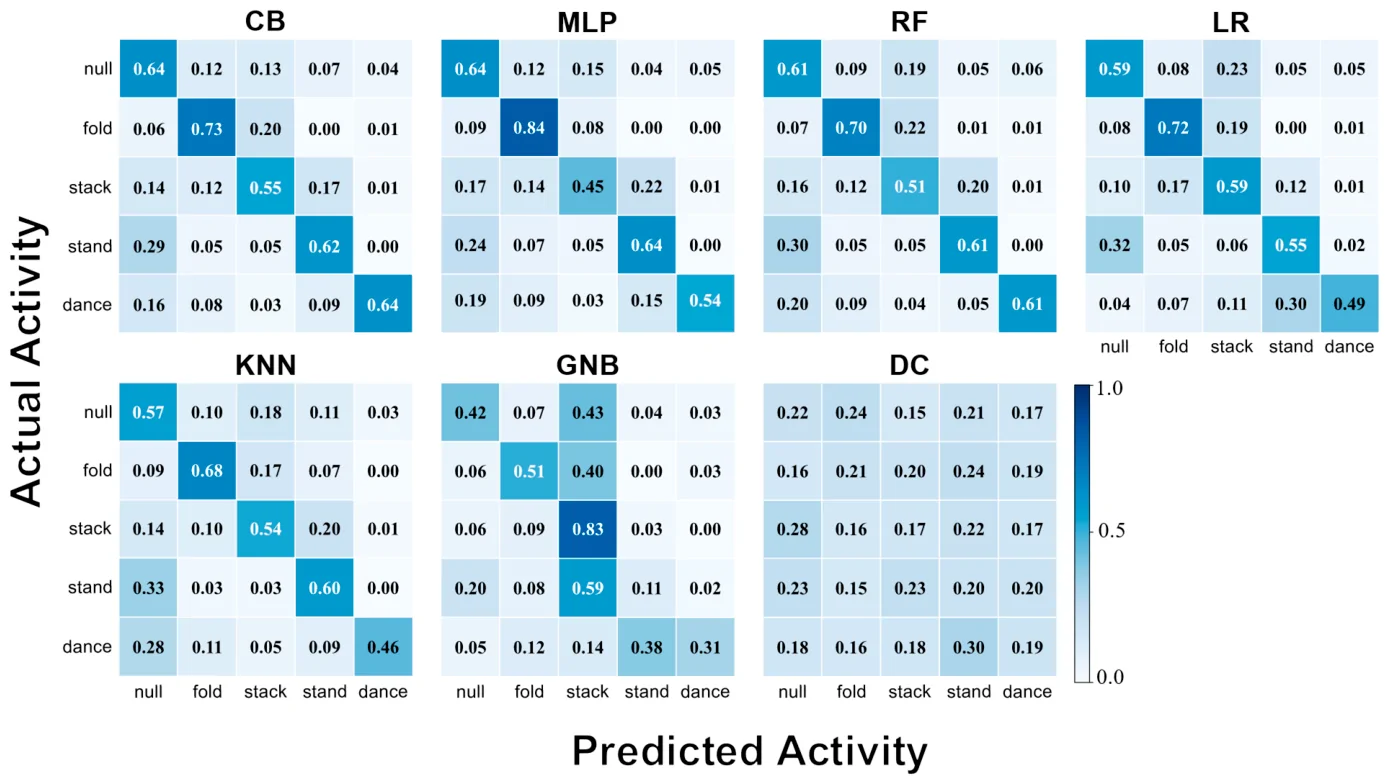
Confusion matrices demonstrating performances of each model (CatBoost, CB; multilayer perceptron, MLP; random forest, RF; logistic regression, LR; k-nearest neighbors, KNN; and gaussian naive bayes, GNB) in correctly classifying activities and null time compared with the dummy classifier (DC) using the expanded feature set. The confusion matrices for 4 of the 6 models have higher values on the diagonal indicating they performed reasonably well as activity recognition algorithms.

Confusion matrices for each trained algorithm of Table 3 show higher values on the diagonal for CatBoost, MLP, RF, LR, and KNN, indicating that all of them performed reasonably well as activity recognition algorithms (i.e., far better than chance, DC; Figure 4). The confusion matrix for the best performer (CatBoost) noticeably shows misclassifications between the “none of the above” (null) activity class and the four experimental activities (most often between null activity and stacking cups), but fewer misclassifications between folding towels and other activities. Across all models, actual (true) null activity windows were often in most confused class pairs (Supplemental Table S1). Similarly, predicted null activity was frequently incorrect. However, class-wise F1 scores suggest stack may have been a harder activity to distinguish (Supplemental Table S2). Misclassifications of the null activity with folding towels (and vice versa) were not very likely.

**Figure 5:**
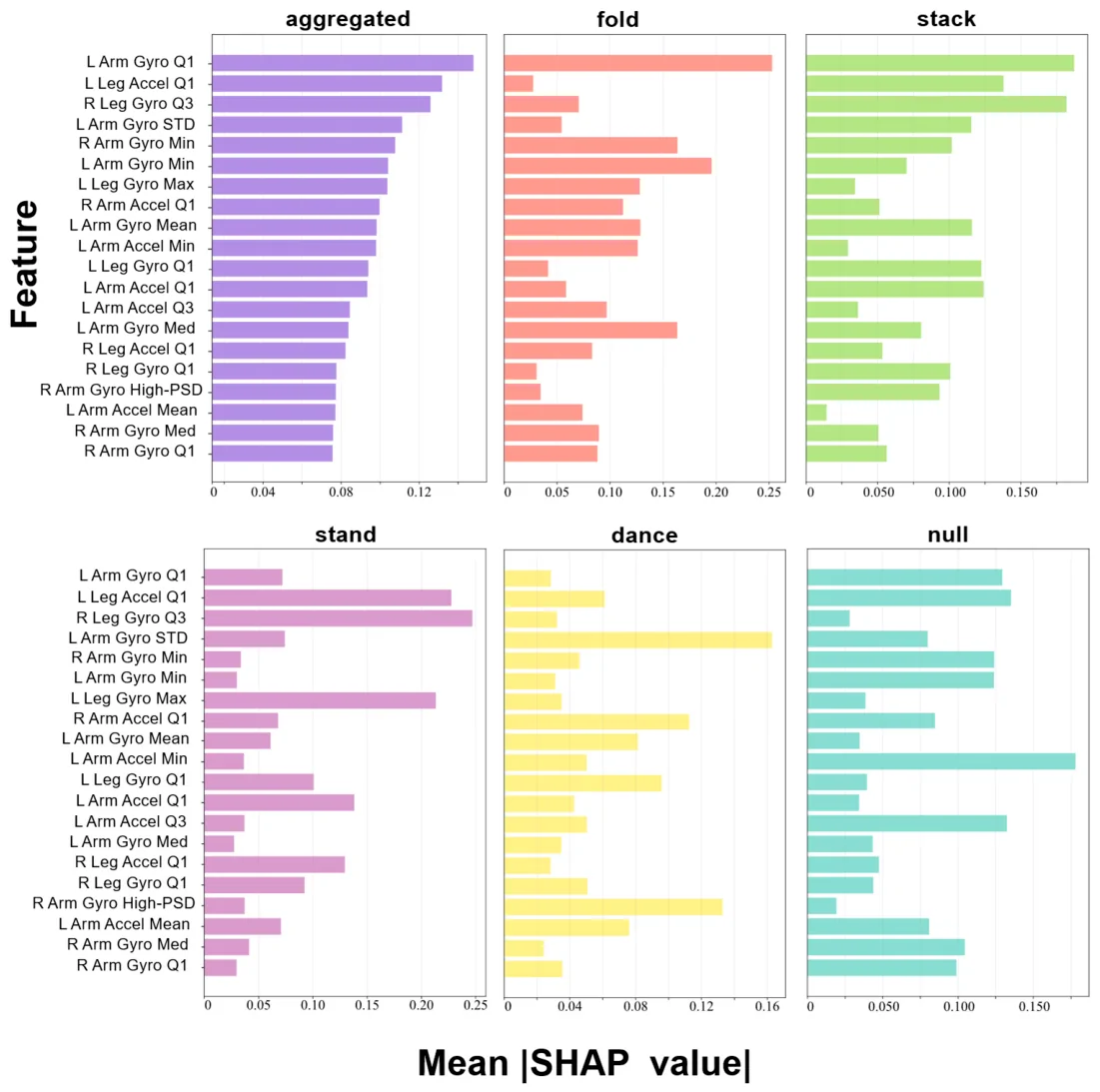
Mean absolute SHAP values from the best trained model (CatBoost) for the expanded features dataset showing the influence of each feature on the model output for the top 20 most influential features.

To evaluate the relative importance of each feature on model output, Shapley Additive Explanation (SHAP) values were determined for the best model (CatBoost; Figure 5). The top five features based on aggregated SHAP values across classes include features from all four limb sensors. Different classes showed different importances for each of these features.

### 3.3. Model Performance with Simplified Feature Set

Using the simplified feature set reduced classification performance across all performance metrics for the trained models (Table 4), albeit only to a modest extent. Here, MLP was the best model when considering accuracy. AUC remained above 0.8 for MLP, CatBoost, and RF. All had very similar 95% CI confidence intervals except for GNB and DC. Statistical testing comparing the best model from expanded features and simplified features shows a statistically significant difference (bootstrap difference test, observed difference: −0.0941 [95% CI: −0.159,−0.031], *p* = 0.002).

### 3.4. Model Performance and Age

To assess the ability of these models in a plausible training scenario, and given the younger age distribution of our population, the participants were divided into an older (age > 10 years) and younger group (age ≤ 10 years), with this age cutoff consistent with literature demonstrating that major improvements in motor coordination, particularly upper-limb coordination, occur throughout childhood and begin to plateau between approximately 8 and 12 years of age [32,33,34]. Using the expanded feature set, models were trained on the older group and evaluated on the younger group (Table 5). ML model performance metrics were largely similar to the expanded feature set performance on groups with mixed ages. Statistical testing comparing the best model from this age split to the best model from the mixed-age group dataset (described above) showed no statistically significant difference (bootstrap difference test, observed difference: −0.0208 [95% CI: −0.0884, 0.0468], *p* = 0.548).

### 3.5. Impact of Transitional Windows

One possible reason for misclassification is poor differentiation between null and other activities due to participants over- or under-extending their activity periods. To assess the extent to which that was the case, windows including and adjacent to transitions between the null and other activities were dropped, and models were trained on the remaining data. Results were very similar to the original expanded feature dataset (Table 6), with statistical testing revealing a lack of significance between best models (bootstrap difference test, observed difference: 8.54 × 10^−3^, [95% CI: −0.0612, 0.0732], *p* = 0.768).

### 3.6. Null Versus Non-Null Classification

**Figure 6:**
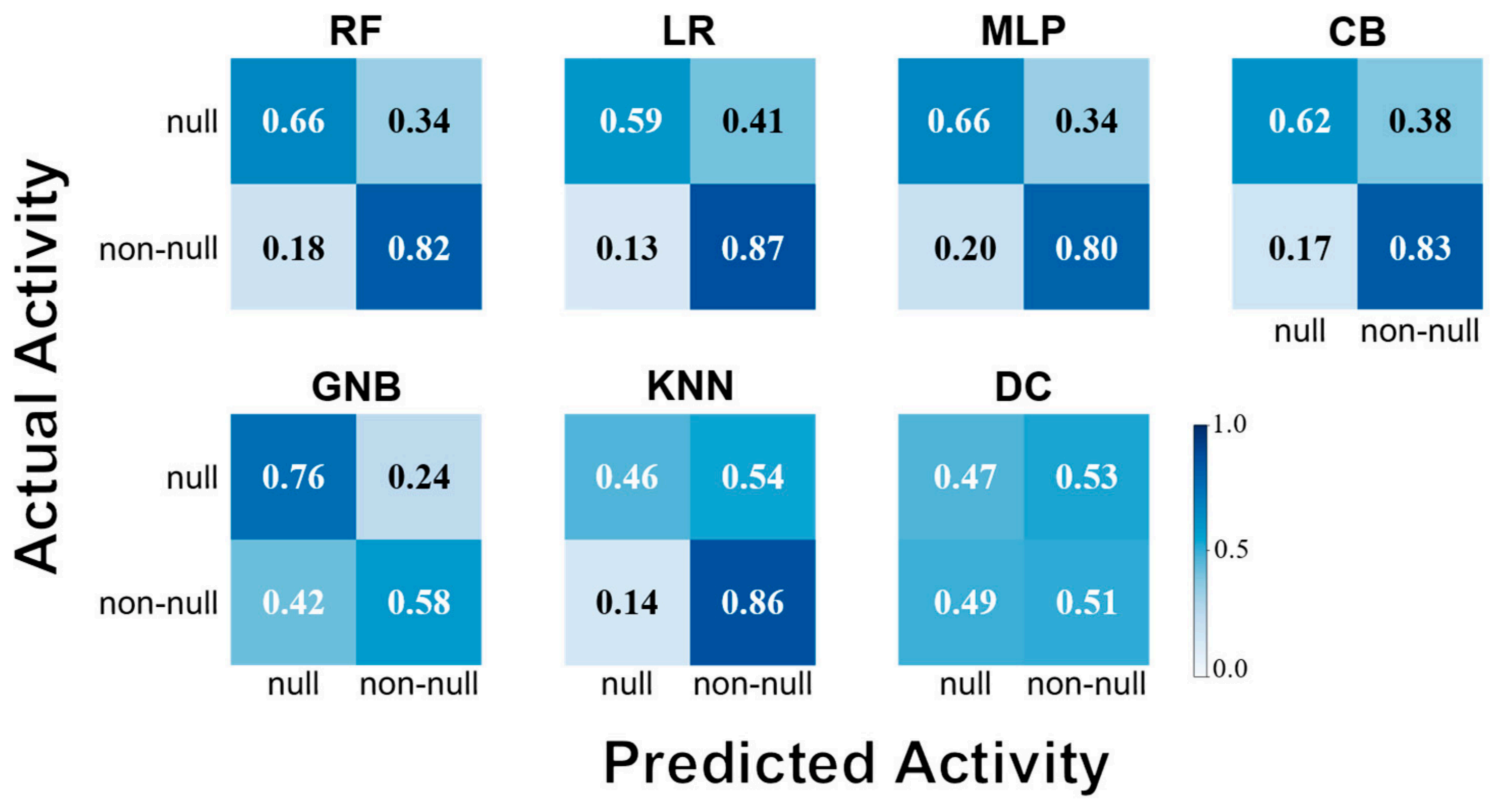
Confusion matrices demonstrating performances of each model (random forest, RF; logistic regression, LR; multilayer perceptron, MLP; CatBoost, CB; gaussian naive bayes, GNB; and k-nearest neighbors, KNN) in correctly classifying null vs. non-null activities compared with the dummy classifier (DC).

**Figure 7:**
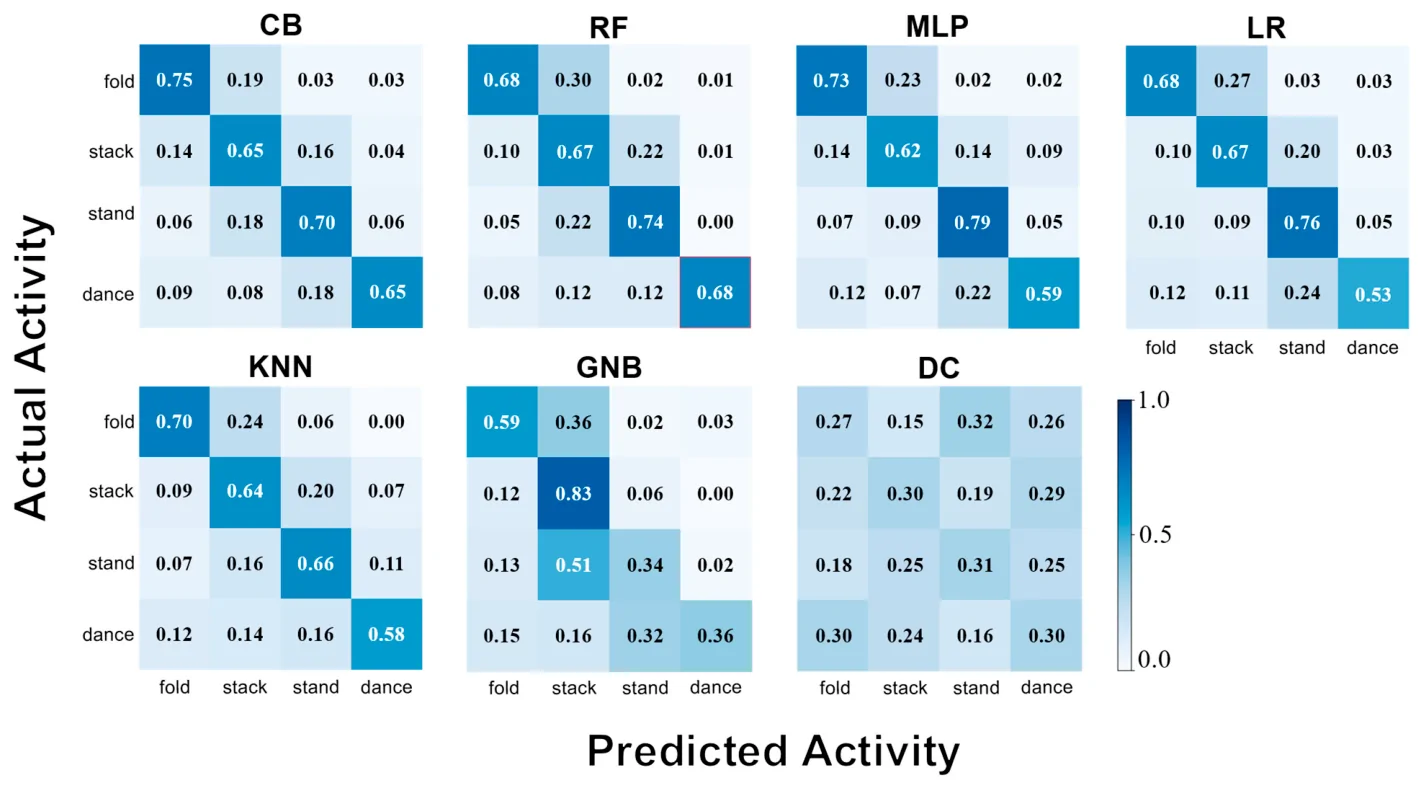
Confusion matrices demonstrating performances of each model (CatBoost, CB; random forest, RF; multilayer perceptron, MLP; logistic regression, LR; k-nearest neighbors, KNN; and gaussian naive bayes, GNB) in correctly classifying activities (without null) compared with the dummy classifier (DC).

Similarly, to determine whether the impact of null misclassification could be reduced, the classification problem was separated into two problems—binary classification between null and non-null activities, and subsequently, differentiation between the four remaining non-null activities. A binary classification model was trained to differentiate null and non-null activities with the expanded feature set, with RF emerging as the best binary classifier (Table 7, Figure 6). In classifying between the four non-null activities, CatBoost was best (Table 8, Figure 7). The binary classification problem showed improved performance relative to the multiclass problem, as did classification excluding null activities. The similar performance with the binary and non-null classification problems suggests that the difficulty comes in both resolving the null class and identifying the correct target activity in a single decision, rather than either boundary alone. A cascaded pipeline, in which null vs. non-null activity is decided prior to activity classification, may be preferable if null periods are frequent in smaller datasets. However, this comes at the cost of layered misclassification if activity is incorrectly determined as null at the binary classification stage. Ultimately, when the binary and non-null models were combined, the resulting model did not show improvement over the models trained on the expanded feature set above (RF + CB, accuracy 0.626 [0.582,0.672], precision 0.622 [0.576,0.666], recall 0.609 [0.563,0.658], F1 Macro 0.614 [0.567,0.660], AUC 0.839 [0.814,0.866]).

### 3.7. Sensor Placement

Choices for the number of sensors and sensor placement have varied considerably in the literature (Table 9). To evaluate the impact of the placement and number of sensors, a permuted list of all possible 15 combinations for sensor placement on the wrist or ankle was created and subsequently used to train sets of MLP, KNN, and DC models using the expanded feature set (Table 10). These models were selected based on the absence of statistical difference between CatBoost and these models in the full set, as well as their lower computational requirements. Best results for both KNN and MLP were seen with dropping the left ankle from the sensor set. Including only single sensors, particularly those in the lower extremity, showed substantial reduction in performance.

### 3.8. Deep Learning

As deep learning has previously been used successfully in HAR and on edge devices, we attempted to employ two deep learning approaches with our dataset (DeepConvLSTM and ResGCNN). Prior to implementation, we benchmarked our performance against the OPPORTUNITY dataset (Table 11), finding that our computing platform was substantially slower than a prior report [38] for both training and classification. In our dataset, we found both deep learning approaches to be much slower than the classical ML techniques (Table 12), while failing to provide performance benefits (Table 13). We also attempted data augmentation for DeepConvLSTM and ResGCNN, but doing so increased train time with only marginal performance benefit (Table 13). We then attempted further optimization of the grid search using the Optuna library, which improved train times (for ResGCNN, total grid search time was 17.2 h down from 76.3 h, and for DeepConvLSTM 4.64 h from 83.2 h), but did not improve performance (Table 13).

### 3.9. Computational Resource Utilization

We determined the time to complete each train and test cycle to evaluate the potential for in situ model training and deployment in devices used for edge computing (Table 12 and Table 14). DeepConvLSTM took the longest time, followed by CatBoost, and GNB the least. The simplified feature set provided substantial training time improvements across all models (except the dummy classifier). Deep learning methods required less memory for inference, but were slower, and had more parameters (Table 14).

We also determined the amount of device storage required to implement each of the ML and deep learning models considered above to evaluate the potential for model deployment in edge computing devices (Table 15). For all models, memory requirements were small, with the largest (RF) requiring less than 7 MB for a model that considers the expanded feature set or about 2 MB for a model that considers the simple feature set. The best performing models from Table 3 (CatBoost and MLP) both consume less than 400 KB with either feature set.

## 4. Discussion

This study investigated the ability of multiple machine learning algorithms (CatBoost, MLP, RF, KNN, LR, GNB, ResGCNN, and DeepConvLSTM) to classify activities based on IMU time series data collected by four sensors fixed to the wrists and ankles during a set of functional activities in a heterogeneous cohort of participants. Across feature sets and model pipelines, all tested algorithms were able to classify activity type with far greater accuracy than that of a dummy classifier that randomly assigned a class, supporting our hypothesis that meaningful activity recognition is feasible in a limited heterogeneous dataset. Contrary to our expectations, however, no single algorithm prevailed as the top performer during our comprehensive evaluation of human activity recognition (HAR) in the given dataset. Together, these findings reinforce that model choices for HAR should be treated as data-specific decisions with careful consideration for how real-world applications (such as the abundance of null activity) may affect algorithm accuracy.

Of the eight different algorithms tested, CatBoost, MLP, and RF had accuracies greater than 0.60, which strongly outperformed the 0.20 accuracy achieved by the dummy classifier. AUCs for CatBoost, MLP, RF, KNN, and LR were all in the excellent range for feature discrimination with the expanded feature set. With the simplified feature set, AUCs were in the excellent range for CatBoost, MLP, and RF. Although the best expanded feature model outperformed the best simple feature model, our results indicate that a lighter feature representation may still be viable if computational resources are constrained. Relative to prior HAR studies using larger or more controlled datasets, our accuracies are more modest (c.f., Table 9).

We originally thought that upper-extremity activities such as folding towels and stacking cups might be more likely to be confused with each other given the sparse input from the lower extremities in these cases; however, this was not borne out. Instead, confusion matrices indicated that errors were most prevalent when differentiating null periods from labeled activities (Figure 4; see also Supplementary Table S1). This pattern is noteworthy because folding towels and stacking cups share overlapping bimanual demands, yet the model separated them more readily than from null activity.

We considered whether movements “bleeding over” into the instructed rest times between activities could have contributed to misclassification between null activity and other activities (see for example the occasional residual activity within the null periods of Figure 2). Model classification results for the best models did not, however, differ significantly when we excluded data windows that included or were adjacent to transitions between the null and other activities (compare Table 3 and Table 6). Another possible contribution to null misclassification could be intermittent resting or other pauses during periods of requested activity, as a pause in movement may appear to be similar to null activity. This might explain why the first activity (folding towels) was less often misclassified as null, as the participants might have been less likely to take a mid-test break as sometimes occurred later in the study. Yet, this interpretation should be treated cautiously, because participation in the target activities was brief (only 60 s per activity) and because later activities, particularly dancing, also may have greater inherent interpersonal variability in movement strategy and style, which could increase misclassification relative to the earlier activities of towel-folding and cup-stacking. The moderate to strong performance of multiple tested pipelines even with the simplified feature set is quite remarkable considering the features used to train and test them were obtained using very short 5 s data windows derived from highly processed kinematic data with very low time-resolution (i.e., an effective sampling interval of 1 s). Despite the difficulty of distinguishing null periods from target activities, we consider inclusion of a null class necessary for translational relevance, because unconstrained real-world recordings are likely to be dominated by intervals that do not correspond to a targeted functional task.

SHAP values from the CatBoost model (Figure 5) indicated that informative features were distributed across all four limb sensors, and model accuracy improved markedly when features from both wrist sensors were included in analysis (Table 10). While CatBoost, a gradient boosting method [39], might be better known for its special handling of categorical data, it has been shown to provide robust performance in human activity recognition [35]. It is also readily interpretable, and feature importance derived from its internal methods provides evidence to support the placement of sensors on all four limbs tested here to improve classifier performance (cf., Figure 5). At the same time, we found that the strongest KNN and MLP results from our sensor dropping analysis were obtained after excluding the left ankle, which may reflect redundancy among lower-limb sensors. Overall, our results align with prior studies indicating that wrist sensors are particularly informative for classifying daily activity [22,39], and that classification accuracy generally improves as the number of sensors increases, albeit with diminishing gains [25,40].

Dataset heterogeneity is likely to occur in clinical populations. We therefore addressed the heterogeneity issue by comparing the generalizability of our models between older (>10 years of age) and younger participants. We found that training on older participants and testing on younger participants yielded performance comparable to mixed-age training and testing, with no significant difference found between the best performing models. Therefore, moderate age-related heterogeneity does not necessarily undermine model generalizability when training and test sets are drawn from healthy populations split based on a 10-year cutoff. This contrasts with prior literature in which transfer learning was required to improve model performance trained on adults and tested on children [20]. Our results do not, however, imply that the models would perform equally well for individuals at more extreme ends of the age range, nor do they guarantee transfer to clinical populations.

Classification performance of the models we described could be improved in a number of different ways. Additional features could be used, such as those obtained using other frequency domain techniques such as wavelet decomposition, which could help differentiate between activities based on complex acceleration profiles [41]. The acceleration and gyroscopic data could be fused during preprocessing to provide additional information about the absolute position and orientation of sensors (and limbs), which has been shown to improve activity recognition accuracy by others [23]. History of previous data epochs could also be included to create time dependencies across sampling windows, which would help identify activities lasting longer than 5 s time periods. Performing clustering on the extracted features prior to classification might also help identify and reduce unnecessary features and improve performance of the classifiers [42]. Likewise, SHAP values can also be used to guide the reduction of features and improvement of classifier performance [24].

CatBoost and the other simpler models may have outperformed deep learning methods in this study due to the limited size of our dataset with its inherent class imbalance (as reflected in overweighting of the null class). These performance issues have been reflected in prior work, with Devi et al. noting poor performance with a bidirectional long short-term memory model compared to CatBoost and other classical ML models on the CMI dataset [35]. Unlike the OPPORTUNITY dataset [7], which had 20 repetitions for each activity, completed by a total of four participants, with six hours of recordings, and where DeepConvLSTM was noted to have an F1 score of 0.930 [38], our study only had a single 60 s repetition of each activity for each of our participants (i.e., about twelve 5 s data windows per target activity per participant). Furthermore, our study only utilized four sensors, versus the 23 available in the OPPORTUNITY dataset. Ultimately, this resulted in our dataset having fewer sensors, fewer activity repetitions, and more variation due to the increased number of participants. When data augmentation was applied to address the smaller dataset size, deep learning methods yielded only marginal accuracy gains (Table 13) while requiring substantially greater training time (Table 14). These efficiency constraints must be considered for translation when using small datasets. We envision a future where wearable technology can promote “precision health” through the use of individually trained activity recognition models. However, as concerns about data security and latency continue, there will continue to be a need to improve efficiency and push more of the training and evaluation of models to wearable devices. In clinical settings, the longer training times of more complex models may not justify incremental increases in performance, particularly in on-device applications. A more practical compromise for small datasets could be offline training with on-device inference, selecting among classical pipelines that strike an appropriate balance between evaluation time and model size.

Our results also demonstrate the importance of comparing multiple models and configurations for a given dataset, as would be anticipated by the “no free lunch” theorem, which states that the best model for a given dataset is not known a priori [43]. The question of whether to use a larger or smaller set of data features needs to balance competing costs and benefits, and the answer will almost certainly be application-dependent. Future efforts should consider the extent to which the inclusion of additional features provides sufficient performance gains to balance the concomitant increase in time both for model training and activity inference, the increase in memory storage space needed to store the added features and code to compute them, and the associated increase in computing power.

### 4.1. Research Gap and Contributions

Prior HAR work has often optimized classifiers, features, or sensor placements in isolation using large laboratory datasets that may not generalize to small or heterogeneous real-world samples. This study contributes a coordinated methodological comparison under data-limited conditions by comprehensively evaluating classical versus deep learning models, expanded versus simple feature sets, age-group transfer, transition window handling, null versus non-null problem structures, and sensor configurations using a community-derived heterogeneous dataset. We show that several classical models can recognize functional activities from bilateral wrist and ankle IMUs with accuracy well above chance; that the expanded features described here yield only modestly improved accuracy; that real-world instances of null activity may hinder algorithm accuracy; and that sensor configuration is likely to be activity dependent. Collectively, these findings provide design guidance for wearable HAR pipelines intended for small-sample applications where inter-individual or task-related heterogeneity is expected.

### 4.2. Limitations and Future Directions

This study has several limitations. First, our sample size of 23 participants is small and consists mostly of Caucasian females. Moreover, although we compared models across “younger” and “older” subgroups, the sample overall remained biased towards younger participants, limiting inference about true older-adult generalization. We also note that our study had fewer repetitions of each activity compared to other datasets like the OPPORTUNITY dataset [7]. Although our data may have higher inter-individual variability, intra-subject variability was perhaps more limited. Even so, accuracy, precision, recall, F1, and AUC were all rather high even considering these limitations. This suggests that our methods were successful in mitigating some effects of sample bias, although further exploration is needed to understand to what extent. Second, methods for preprocessing data could have been subject to error. Specifically, we acknowledge some risk of data class mislabeling, especially during the identification of the start and end times for activities, which was completed manually and was guided by protocol comments and deviation notes created during data collection. The limited difference in model performance when removing transition windows might suggest that activity start- and end-time labeling errors alone are unlikely to explain most misclassifications.

Our study carefully considered the translational relevance of our methods to real-world clinical applications. Although data were collected in a community setting and tasks were designed to allow inter-individual variability resembling everyday behavior, the protocol was not truly “uncontrolled” because participants were guided through the series of timed activities by a member of the study team. Participants completed activities in a public setting with wearable sensors they would not otherwise have been wearing, which together may alter natural movement. Fully eliminating observer effects using wearables is difficult; however, other approaches, such as participant-reported task labeling by Stojchevska et al. (2023) [11], may allow for better approximation of real-world behavior and should be considered to improve translational relevance of HAR.

## 5. Conclusions

In this study, we evaluated the ability of multiple machine learning algorithms to classify functional activities using IMU-derived time series data collected from sensors placed on the bilateral wrists and ankles. All tested models outperformed the dummy classifier, demonstrating that meaningful activity classification can be achieved even with short-duration data segments and a relatively small, heterogeneous dataset.

Contrary to our original expectations, our findings highlight that no single algorithm unambiguously dominated all other tested models across a relatively wide range of performance metrics for both a simplified and an expanded set of features. This outcome demonstrates the importance of systematically evaluating multiple models and feature sets for a given application. Relative to many lab-based benchmarks that report near-ceiling accuracy on stereotyped activities in homogeneous cohorts, our community-derived accuracies are more modest. Nevertheless, and despite modest overall accuracy, the similar levels of performance across the top three models in this study suggest that across a broad range of implementations, wearable IMU systems can demonstrate feasibility in community-based conditions with practical sensor placement configurations.

## Figures and Tables

**Table 1:** Participant demographics as percent of total or median with interquartile range.

Female	69.6%
Race: White	95.7%
Age (y)	12 [10.5,26.5]
Height (in)	60 [56,64.5]
Weight (lb)	103 [80,140]

**Table 2:** Class distribution in the complete dataset.

Model	Samples (*n*)	Percent of Total
Null	417	30.4%
Fold	285	20.7%
Stack	251	18.3%
Stand	219	15.9%
Dance	202	14.7%

**Table 3:** Expanded features—ML model performance on test dataset.

Mode	Accuracy	Precision	Recall	F1 Macro	AUC
CatBoost	0.644 [0.600,0.686]	0.657 [0.613,0.698]	0.637 [0.591,0.681]	0.643 [0.597,0.685]	0.847 [0.819,0.875]
MLP	0.642 [0.603,0.684]	0.650 [0.606,0.692]	0.623 [0.578,0.666]	0.629 [0.580,0.671]	0.829 [0.798,0.857]
RF	0.613 [0.573,0.657]	0.629 [0.587,0.671]	0.606 [0.564,0.653]	0.613 [0.571,0.656]	0.843 [0.817,0.869]
LR	0.598 [0.556,0.644]	0.613 [0.569,0.660]	0.590 [0.547,0.638]	0.591 [0.546,0.633]	0.818 [0.792,0.844]
KNN	0.577 [0.533,0.621]	0.612 [0.569,0.656]	0.569 [0.526,0.614]	0.576 [0.531,0.620]	0.807 [0.777,0.835]
GNB	0.427 [0.385,0.471]	0.492 [0.445,0.541]	0.437 [0.402,0.476]	0.410 [0.368,0.452]	0.780 [0.756,0.804]
DC	0.201 [0.167,0.239]	0.200 [0.165,0.235]	0.197 [0.162,0.233]	0.195 [0.161,0.231]	0.500 [0.500,0.500]

**Table 4:** Simple features—ML model performance on test dataset.

Model	Accuracy	Precision	Recall	F1 Macro	AUC
MLP	0.567 [0.525,0.615]	0.577 [0.529,0.629]	0.543 [0.499,0.593]	0.547 [0.501,0.598]	0.800 [0.771,0.829]
CatBoost	0.550 [0.506,0.594]	0.563 [0.517,0.608]	0.542 [0.498,0.592]	0.545 [0.501,0.590]	0.832 [0.807,0.858]
RF	0.542 [0.496,0.586]	0.555 [0.508,0.601]	0.535 [0.487,0.582]	0.536 [0.489,0.580]	0.797 [0.768,0.825]
KNN	0.517 [0.475,0.563]	0.552 [0.508,0.596]	0.511 [0.466,0.558]	0.510 [0.465,0.554]	0.750 [0.719,0.782]
LR	0.508 [0.464,0.556]	0.534 [0.487,0.586]	0.498 [0.454,0.548]	0.497 [0.451,0.545]	0.791 [0.764,0.817]
GNB	0.431 [0.387,0.475]	0.510 [0.461,0.560]	0.450 [0.407,0.490]	0.428 [0.384,0.467]	0.761 [0.735,0.789]
DC	0.201 [0.167,0.239]	0.200 [0.165,0.235]	0.197 [0.162,0.233]	0.195 [0.161,0.231]	0.500 [0.500,0.500]

**Table 5:** Expanded feature set, trained on older participants (age > 10 years), evaluated on younger participants (age ≤ 10 years).

Model	Accuracy	Precision	Recall	F1 Macro	AUC
CatBoost	0.624 [0.571,0.674]	0.628 [0.574,0.682]	0.608 [0.554,0.660]	0.614 [0.559,0.664]	0.844 [0.813,0.873]
RF	0.606 [0.553,0.656]	0.609 [0.554,0.662]	0.596 [0.540,0.648]	0.599 [0.542,0.648]	0.830 [0.798,0.862]
LR	0.565 [0.509,0.618]	0.564 [0.507,0.622]	0.544 [0.488,0.596]	0.546 [0.487,0.600]	0.797 [0.764,0.831]
GNB	0.529 [0.482,0.582]	0.545 [0.491,0.601]	0.542 [0.493,0.594]	0.513 [0.463,0.564]	0.766 [0.729,0.801]
MLP	0.541 [0.494,0.594]	0.545 [0.492,0.601]	0.537 [0.485,0.594]	0.538 [0.485,0.592]	0.791 [0.755,0.824]
KNN	0.535 [0.482,0.591]	0.529 [0.474,0.589]	0.518 [0.462,0.576]	0.521 [0.464,0.578]	0.791 [0.758,0.827]
DC	0.182 [0.141,0.227]	0.177 [0.137,0.220]	0.180 [0.139,0.225]	0.175 [0.135,0.216]	0.500 [0.500,0.500]

**Table 6:** Model performance with transition windows removed (expanded feature dataset).

Model	Accuracy	Precision	Recall	F1 Macro	AUC
LR	0.656 [0.609,0.705]	0.686 [0.642,0.730]	0.648 [0.602,0.701]	0.652 [0.603,0.702]	0.840 [0.809,0.870]
CatBoost	0.653 [0.603,0.702]	0.672 [0.626,0.716]	0.637 [0.585,0.686]	0.648 [0.596,0.695]	0.874 [0.847,0.899]
RF	0.639 [0.590,0.689]	0.671 [0.627,0.714]	0.629 [0.580,0.679]	0.640 [0.592,0.685]	0.868 [0.839,0.895]
MLP	0.639 [0.590,0.689]	0.660 [0.611,0.707]	0.623 [0.572,0.674]	0.627 [0.576,0.678]	0.846 [0.817,0.876]
KNN	0.617 [0.565,0.667]	0.641 [0.595,0.685]	0.604 [0.551,0.654]	0.611 [0.557,0.659]	0.825 [0.793,0.856]
GNB	0.468 [0.413,0.526]	0.559 [0.499,0.620]	0.479 [0.430,0.529]	0.460 [0.406,0.516]	0.788 [0.762,0.816]
DC	0.201 [0.157,0.242]	0.197 [0.155,0.239]	0.197 [0.156,0.242]	0.195 [0.154,0.236]	0.500 [0.500,0.500]

**Table 7:** Performance metrics for the binary classification of null-vs-non-null.

Model	Accuracy	Precision	Recall	F1 Macro	AUC
RF	0.789 [0.749,0.824]	0.732 [0.691,0.776]	0.743 [0.700,0.784]	0.737 [0.695, 0.777]	0.809 [0.766,0.853]
LR	0.774 [0.734,0.814]	0.749 [0.702,0.796]	0.733 [0.685,0.776]	0.734 [0.693,0.784]	0.800 [0.756,0.844]
MLP	0.766 [0.726,0.803]	0.717 [0.674,0.759]	0.732 [0.688,0.776]	0.723 [0.680,0.764]	0.810 [0.765,0.854]
CatBoost	0.759 [0.720,0.795]	0.721 [0.675,0.764]	0.723 [0.677,0.766]	0.722 [0.677,0.762]	0.807 [0.763,0.851]
GNB	0.738 [0.699,0.778]	0.640 [0.601,0.681]	0.667 [0.623,0.711]	0.620 [0.573,0.664]	0.742 [0.690,0.792]
KNN	0.632 [0.586,0.674]	0.684 [0.631,0.733]	0.659 [0.611,0.706]	0.667 [0.616,0.714]	0.759 [0.710,0.805]
DC	0.496 [0.452,0.538]	0.490 [0.450,0.530]	0.488 [0.440,0.535]	0.471 [0.427,0.515]	0.500 [0.500,0.500]

**Table 8:** Performance metrics for classification of non-null activities.

Model	Accuracy	Precision	Recall	F1 Macro	AUC
CatBoost	0.696 [0.645,0.743]	0.702 [0.652,0.748]	0.689 [0.637,0.739]	0.691 [0.637,0.738]	0.872 [0.838,0.901]
RF	0.696 [0.648,0.743]	0.729 [0.684,0.770]	0.689 [0.635,0.738]	0.695 [0.644,0.740]	0.869 [0.837,0.898]
MLP	0.690 [0.639,0.734]	0.697 [0.646,0.745]	0.686 [0.634,0.734]	0.687 [0.633,0.735]	0.876 [0.845,0.904]
LR	0.663 [0.609,0.711]	0.679 [0.625,0.728]	0.657 [0.601,0.708]	0.657 [0.600,0.705]	0.839 [0.805,0.871]
KNN	0.651 [0.600,0.701]	0.658 [0.607,0.706]	0.645 [0.592,0.695]	0.646 [0.592,0.695]	0.818 [0.780,0.853]
GNB	0.525 [0.469,0.582]	0.599 [0.542,0.654]	0.532 [0.478,0.582]	0.516 [0.458,0.571]	0.798 [0.763,0.829]
DC	0.293 [0.242,0.340]	0.294 [0.244,0.342]	0.295 [0.244,0.342]	0.292 [0.242,0.338]	0.500 [0.500,0.500]

**Table 9:** Comparison of model performance with similar prior HAR studies.

Study	Dataset	(Number of) Sensors	Window Size (s)	Classifiers	Accuracy	Model Size (KB)
Zhou et al. 2025 [30]	WISDM	(2) smartphone and unilateral wrist	5 (multiple tested)	1D CNN, 2D CNN, DeepConv LSTM, Res-GCNN	0.96–0.98	136.5–633.47
Devi et al. 2026 [35]	CMI	(1) unilateral wrist	Not provided	CB, XGB, LGBM, ExtraTrees, BiLSTM, Transformer	0.81–1.00	Not provided
Rahman et al. 2020 [36]	Public dataset [37]	(1) smartphone	2.56	XGB, LGBM, GB, CB, AB	0.80–0.96	Not provided
Current study	Community derived dataset	(4) bilateral wrists and ankles	5	MLP, RF, KNN, LR, CB, GNB, DeepConvLSTM, Res-GCNN	up to 0.789	606–2047

Abbreviations: CNN: convolutional neural network; CB: CatBoost; XGB: eXtreme gradient boosting; LGBM: light gradient boosting; GB: gradient boosting; AB: AdaBoost; MLP: multilayer perceptron; RF: random forest; KNN: k-nearest neighbors; LR: logistic regressor; GNB: gaussian naïve bayes.

**Table 10:** Balanced accuracy of various models on cross validation set removing limbs from dataset.

Right Ankle	Left Ankle	Right Wrist	Left Wrist	DC	KNN	MLP
✓	-	-	-	0.185	0.429	0.517
-	✓	-	-	0.185	0.500	0.515
-	-	✓	-	0.185	0.580	0.603
-	-	-	✓	0.185	0.572	0.607
✓	✓	-	-	0.185	0.464	0.526
✓	-	-	✓	0.185	0.666	0.688
✓	-	✓	-	0.185	0.651	0.700
-	✓	-	✓	0.185	0.642	0.678
-	✓	✓	-	0.185	0.633	0.666
-	-	✓	✓	0.185	0.620	0.631
✓	✓	-	✓	0.185	0.646	0.672
✓	✓	✓	-	0.185	0.633	0.677
✓	-	✓	✓	0.185	0.693	0.713
-	✓	✓	✓	0.185	0.677	0.688
✓	✓	✓	✓	0.185	0.687	0.703

**Table 11:** Comparative model performance on OPPORTUNITY dataset.

	Training Time (min)	Classification (s)
Present Study	2127.5	306
Ordonez & Roggen [38]	340.3	6.68

**Table 12:** Training time per grid element in seconds.

Model	Expanded Features	Simple Features	Raw Data
DC	0.061	0.032	-
GNB	0.064	0.045	-
KNN	0.077	0.059	-
MLP	0.165	0.107	-
LR	0.324	0.130	-
RF	7.879	1.619	-
CatBoost	23.032	6.296	-
DeepConvLSTM	-	-	689
ResGCNN	-	-	1716
DeepConvLSTM (Augmented Dataset)	-	-	1873

**Table 13:** Performance metrics for deep learning methods.

Model	Augmentation	Optuna	Accuracy	Precision	Recall	F1 Macro	AUC
ResGCNN	Yes	Yes	0.487 [0.457,0.514]	0.495 [0.462,0.523]	0.476 [0.444,0.523]	0.478 [0.445,0.505]	0.769 [0.750,0.787]
ResGCNN	Yes	No	0.446 [0.428,0.466]	0.702 [0.652,0.748]	0.689 [0.637,0.739]	0.691 [0.637,0.738]	0.872 [0.838,0.901]
DeepConvLSTM	Yes	Yes	0.441 [0.428,0.456]	0.440 [0.424,0.457]	0.409 [0.396,0.423]	0.409 [0.395,0.425]	0.725 [0.716,0.735]
DeepConvLSTM	Yes	No	0.464 [0.450,0.478]	0.729 [0.684,0.770]	0.689 [0.635,0.738]	0.695 [0.644,0.740]	0.869 [0.837,0.898]
DeepConvLSTM	No	No	0.443 [0.429,0.457]	0.697 [0.646,0.745]	0.686 [0.634,0.734]	0.687 [0.633,0.735]	0.876 [0.845,0.904]

**Table 14:** Computer resource utilization metrics for models trained with the expanded features or augmented raw data.

Model	Parameters	FLOPs per Sample Inference (KFLOPs)	Inference Throughput (Sample/s)	Inference CPU Usage (Mean; %)	Inference Memory Usage (Max; MB)
MLP	14,205	28	258,720	100	1744
KNN	112,340	225	6285	120	1743
LR	415	1	270,073	93	1743
RF	69,550	3	12,164	102	1743
GNB	110	0	307,174	103	1743
DC	0	0	371,391	103	1743
CatBoost	9300	14	148,157	104	1743
ResGCNN	506,701	100,577	500	382	1189
DeepConvLSTM	149,509	299	5565	385	719

**Table 15:** Model size (KB) on disk.

Model	Expanded Features	Simple Features	Raw Data
DC	4.5	2	-
GNB	15	11	-
LR	76	3	-
MLP	335	197	-
CatBoost	365	192	-
DeepConvLSTM	-	-	606
KNN	590	169	-
ResGCNN	-	-	2047
RF	7100	2200	-

## Data Availability

The raw data and code supporting the conclusions of this article will be made available by the authors on request.

## Supplementary Materials

The following supporting information can be downloaded at https://www.mdpi.com/article/10.3390/s26175357/s1: Table S1: Top 5 most confused pairs for each model; Table S2: Class-wise F1 scores with 95% CI in the test expanded feature set; Table S3: Demographics and physical characteristics of participants; and File S1: Hyperparameters and Architecture.

## Abbreviations

The following abbreviations are used in this manuscript:

ML	Machine Learning
IMU	Inertial Measurement Unit
RMS	Root Mean Square
MLP	Multilayer Perceptron
RF	Random Forest
KNN	K-Nearest Neighbors
LR	Logistic Regressor
GNB	Gaussian Naïve Bayes
RUS	Random Undersampling
SMOTE	Synthetic Minority Oversampling Technique
PCA	Principal Component Analysis
SFM	Select-From-Model
AUC	Area Under the Receiver Operating Characteristic Curve

## References

[1] Wei S., Wu Z. (2023). The Application of Wearable Sensors and Machine Learning Algorithms in Rehabilitation Training: A Systematic Review. Sensors.

[2] Boukhennoufa I., Zhai X., Utti V., Jackson J., McDonald-Maier K.D. (2022). Wearable sensors and machine learning in post-stroke rehabilitation assessment: A systematic review. Biomed. Signal Process. Control.

[3] Rast F.M., Labruyère R. (2020). Systematic review on the application of wearable inertial sensors to quantify everyday life motor activity in people with mobility impairments. J. Neuroeng. Rehabil..

[4] Sabry F., Eltaras T., Labda W., Alzoubi K., Malluhi Q. (2022). Machine Learning for Healthcare Wearable Devices: The Big Picture. J. Healthc. Eng..

[5] Nguyen B., Coelho Y., Bastos T., Krishnan S. (2021). Trends in human activity recognition with focus on machine learning and power requirements. Mach. Learn. Appl..

[6] Hou C. A study on IMU-based human activity recognition using deep learning and traditional machine learning. Proceedings of the 2020 5th International Conference on Computer and Communication Systems, ICCCS 2020.

[7] Roggen D., Calatroni A., Rossi M., Holleczek T., Forster K., Troster G., Lukowicz P., Bannach D., Pirkl G., Ferscha A. Collecting complex activity datasets in highly rich networked sensor environments. Proceedings of the INSS 2010—7th International Conference on Networked Sensing Systems.

[8] Reiss A. (2012). PAMAP2 Physical Activity Monitoring [Dataset].

[9] Weiss G.M. (2019). WISDM Smartphone and Smartwatch Activity and Biometrics Dataset.

[10] Reyes-Ortiz J., Anguita D., Ghio A., Oneto L., Parra X. (2013). Human Activity Recognition Using Smartphones [Dataset].

[11] Stojchevska M., De Brouwer M., Courteaux M., Ongenae F., Van Hoecke S. (2023). From Lab to Real World: Assessing the Effectiveness of Human Activity Recognition and Optimization through Personalization. Sensors.

[12] Tavasoli S., Tavasoli M., Shojaeefard M., Farahmand F. (2023). Analysis of cerebral palsy gait based on movement primitives. Clin. Biomech..

[13] Delmas S., Tiwari A., Tseng H.Y., Poisson S.N., Diehl M., Lodha N. (2025). Amplified Intraindividual Variability in Motor Performance in Stroke Survivors: Links to Cognitive and Clinical Outcomes. Brain Behav..

[14] Cavanagh S.K., Gochyyev P., Nayeem R., Dusang A.N., Hamilton T., DiCarlo J.A., Kautz S.A., Sternad D., Walsh C., Hochberg L. (2025). Trial-By-Trial Variation In Upper Extremity Movement Smoothness After Acute Stroke Relates To Clinical Assessments And Corticospinal Tract Injury. Neurorehabilit. Neural Repair.

[15] Chiudo M.M., Bet P., Costa G.F., Simões M.D.S.M.P., Ponti M.A., Dourado V.Z., Castro P.C. (2022). Mapping features and patterns of accelerometry data on human movement in different age groups and associated health problems: A cross-sectional study. Exp. Gerontol..

[16] Mikos V., Yen S.-C., Tay A., Heng C.-H., Chung C.L.H., Liew S.H.X., Tan D.M.L., Au W.L. (2018). Regression analysis of gait parameters and mobility measures in a healthy cohort for subject-specific normative values. PLoS ONE.

[17] Zhang X., Li F., Hobbelen H.S., van Munster B.C., Lamoth C.J. (2025). Gait parameters and daily physical activity for distinguishing pre-frail, frail, and non-frail older adults: A scoping review. J. Nutr. Health Aging.

[18] Stewart T., Narayanan A., Hedayatrad L., Neville J., Mackay L., Duncan S. (2018). A dual-accelerometer system for classifying physical activity in children and adults. Med. Sci. Sports Exerc..

[19] Trost S.G., Cliff D.P., Ahmadi M.N., Van Tuc N., Hagenbuchner M. (2018). Sensor-enabled Activity Class Recognition in Preschoolers: Hip versus Wrist Data. Med. Sci. Sports Exerc..

[20] Li J., Kang P., Tan T., Shull P.B. (2022). Transfer Learning Improves Accelerometer-Based Child Activity Recognition via Subject-Independent Adult-Domain Adaption. IEEE J. Biomed. Health Inform..

[21] Alanazi M., Aldahr R.S., Ilyas M. (2024). Human Activity Recognition through Smartphone Inertial Sensors with ML Approach. Eng. Technol. Appl. Sci. Res..

[22] Kwon Y., Ho Y., Heo J.-H., Jeon H.-M., Kim J., Eom G.-M. (2018). Optimization of Sensor Placement Combinations and Classification Thresholds for the Accelerometer-Based Activity Recognition. J. Med. Imaging Health Inform..

[23] Chen J., Sun Y., Sun S. (2021). Improving human activity recognition performance by data fusion and feature engineering. Sensors.

[24] Gebreyesus Y., Dalton D., Nixon S., De Chiara D., Chinnici M. (2023). Machine Learning for Data Center Optimizations: Feature Selection Using Shapley Additive exPlanation (SHAP). Future Internet.

[25] Gao L., Bourke A.K., Nelson J. (2014). Evaluation of accelerometer based multi-sensor versus single-sensor activity recognition systems. Med. Eng. Phys..

[26] Shoaib M., Bosch S., Incel O.D., Scholten H., Havinga P.J.M. (2016). Complex human activity recognition using smartphone and wrist-worn motion sensors. Sensors.

[27] Bailey R.R., Klaesner J.W., Lang C.E. (2014). An accelerometry-based methodology for assessment of real-world bilateral upper extremity activity. PLoS ONE.

[28] Najafi B., Aminian K., Paraschiv-Ionescu A., Loew F., Büla C.J., Robert P. (2003). Ambulatory system for human motion analysis using a kinematic sensor: Monitoring of daily physical activity in the elderly. IEEE Trans. Biomed. Eng..

[29] Bai L. Time-Frequency Analysis of Upper Limb Motion Based on Inertial Sensors. Proceedings of the 2021 32nd Irish Signals and Systems Conference, ISSC 2021.

[30] Zhou H., Zhang X., Feng Y., Zhang T., Xiong L. (2025). Efficient human activity recognition on edge devices using DeepConv LSTM architectures. Sci. Rep..

[31] Liao T., Zhao J., Liu Y., Ivanov K., Xiong J., Yan Y. Deep Transfer Learning with Graph Neural Network for Sensor-Based Human Activity Recognition. Proceedings of the 2022 IEEE International Conference on Bioinformatics and Biomedicine, BIBM 2022.

[32] Schneiberg S., Sveistrup H., McFadyen B., McKinley P., Levin M.F. (2002). The development of coordination for reach-to-grasp movements in children. Exp. Brain Res..

[33] Gasser T., Rousson V., Caflisch J., Jenni O.G. (2010). Development of motor speed and associated movements from 5 to 18 years. Dev. Med. Child Neurol..

[34] Kuhtz-Buschbeck J.P., Stolze H., Jöhnk K., Boczek-Funcke A., Illert M. (1998). Development of prehension movements in children: A kinematic study. Exp. Brain Res..

[35] Devi H., Kumar P., Govindarajan V., Kumar S., Lohano R., Hitesh H., Shiwlani A. (2026). A Comparative Study of Classical Machine Learning and Deep Learning Approaches for Human Behavior Detection Using Multisensor Data. IEEE Access.

[36] Rahman S., Irfan M., Raza M., Ghori K.M., Yaqoob S., Awais M. (2020). Performance analysis of boosting classifiers in recognizing activities of daily living. Int. J. Environ. Res. Public Health.

[37] Anguita D., Ghio A., Oneto L., Parra X., Reyes-Ortiz J.L. A public domain dataset for human activity recognition using smartphones. Proceedings of the ESANN 2013 Proceedings, 21st European Symposium on Artificial Neural Networks, Computational Intelligence and Machine Learning.

[38] Ordóñez F.J., Roggen D. (2016). Deep convolutional and LSTM recurrent neural networks for multimodal wearable activity recognition. Sensors.

[39] Prokhorenkova L., Gusev G., Vorobev A., Dorogush A.V., Gulin A. (2018). Catboost: Unbiased boosting with categorical features. Advances in Neural Information Processing Systems, Proceedings of the 32nd International Conference on Neural Information Processing Systems, Montreal, Canada, 2018.

[40] Tang Q., John D., Thapa-Chhetry B., Arguello D.J., Intille S. (2020). Posture and Physical Activity Detection: Impact of Number of Sensors and Feature Type. Med. Sci. Sports Exerc..

[41] Preece S.J., Goulermas J.Y., Kenney L.P.J., Howard D., Meijer K., Crompton R. (2009). Activity identification using body-mounted sensors—A review of classification techniques. Physiol. Meas..

[42] Alapati Y.K., Sindhu K. (2016). Combining Clustering with Classification: A Technique to Improve Classification Accuracy. Int. J. Comput. Sci. Eng..

[43] Wolpert D.H. (1996). The Lack of a Priori Distinctions between Learning Algorithms. Neural Comput..

