# Supplementary material for: A Comparison of Machine Learning Approaches to Activity Classification Using IMU Data Collected in a Community-Based Setting
Source: Sensors (Basel). 2026 Aug 25;26(17):5357. doi: 10.3390/s26175357 (PMC13568233; doi:10.3390/s26175357)
Supplement: Supplementary file 1 [file sensors-26-05357-s001.zip › Table S1 Top 5 Most Confused Pairs for Each Model.pdf]

Supplemental Table S1: Top 5 Most Confused Pairs for Each Model.

Model	True Activity	Predicted Activity	Percentage of True Class
CatBoost	stand	null	28.7
CatBoost	fold	stack	20.0
CatBoost	stack	stand	17.4
CatBoost	null	stack	12.6
CatBoost	null	fold	11.9
MLP	stand	null	24.1
MLP	stack	stand	21.7
MLP	dance	null	18.9
MLP	null	stack	14.7
MLP	null	fold	11.9
RF	stand	null	29.9
RF	fold	stack	21.9
RF	stack	stand	20.3
RF	dance	null	20.3
RF	null	stack	18.9
LR	stand	null	32.2
LR	dance	stand	29.7
LR	null	stack	23.1
LR	fold	stack	19.1
LR	stack	fold	17.4
KNN	stand	null	33.3
KNN	dance	null	28.4
KNN	null	stack	18.2
KNN	fold	stack	17.1
KNN	null	stand	11.2
GNB	stand	stack	58.6
GNB	null	stack	43.4
GNB	fold	stack	40.0
GNB	dance	stand	37.8
GNB	stand	null	19.5
DC	null	fold	24.5
DC	fold	stand	23.8
DC	null	stand	21.0
DC	null	dance	17.5
DC	null	stack	15.4
