# Supplementary material for: A Comparison of Machine Learning Approaches to Activity Classification Using IMU Data Collected in a Community-Based Setting
Source: Sensors (Basel). 2026 Aug 25;26(17):5357. doi: 10.3390/s26175357 (PMC13568233; doi:10.3390/s26175357)
Supplement: Supplementary file 1 [file sensors-26-05357-s001.zip › Table S2 Class-wise F1 Scores with 95% CI in the Test Expanded Feature Set.pdf]

Supplemental Table S2. Classwise F1 Scores with 95% CI in the Test Expanded Feature Set.

Model	Null	Fold	Stack	Stand	Dance
CatBoost	0.639 [0.569,0.701]	0.710 [0.639,0.775]	0.500 [0.403,0.593]	0.635 [0.545,0.714]	0.729 [0.642,0.810]
RF	0.602 [0.533,0.667]	0.695 [0.624,0.763]	0.435 [0.331,0.532]	0.639 [0.546,0.723]	0.692 [0.595,0.779]
MLP	0.632 [0.567,0.694]	0.755 [0.690,0.816]	0.459 [0.350,0.561]	0.640 [0.554,0.715]	0.656 [0.552,0.753]
LR	0.620 [0.552,0.687]	0.714 [0.646,0.776]	0.466 [0.375,0.554]	0.558 [0.466,0.652]	0.595 [0.479,0.696]
KNN	0.558 [0.488,0.621]	0.679 [0.604,0.744]	0.471 [0.376,0.563]	0.568 [0.480,0.653]	0.602 [0.492,0.707]
GNB	0.513 [0.430,0.587]	0.565 [0.481,0.650]	0.392 [0.324,0.459]	0.150 [0.067,0.241]	0.430 [0.309,0.542]
DC	0.255 [0.180,0.329]	0.222 [0.145,0.302]	0.153 [0.076,0.234]	0.173 [0.103,0.244]	0.173 [0.095,0.253]
