# Supplementary material for: A Comparison of Machine Learning Approaches to Activity Classification Using IMU Data Collected in a Community-Based Setting
Source: Sensors (Basel). 2026 Aug 25;26(17):5357. doi: 10.3390/s26175357 (PMC13568233; doi:10.3390/s26175357)
Supplement: Supplementary file 1 [file sensors-26-05357-s001.zip › Table S3 Demographics and Physical Characteristics of Participants.pdf]

Supplemental Table S3. Demographics and Physical Characteristics of Participants.

Age	Height	Weight	Sex	Race
11	60	100	male	white
12	60	85	male	white
3	42	35	female	white
4	40	52	female	white
29	63	130	female	white
21	60	110	female	white
68	67	145	female	white
59	65	135	female	white
10	55	70	male	white
8	53	65	male	white
5	42	40	female	white
65	65	190	female	white
11	65	115	female	white
13	64	111	female	white
24	60	103	female	white/asian
10	57	80	female	white
34	62	160	female	white
23	69	145	female	white
11	54	80	male	white
49	69	165	female	white
11	59	89	male	white
11	62	160	male	white
12	60	87	female	white
